# 

**Published:** 1918-01-12

**Authors:** 


### CONTENTS.

| | PAGE |
|---|---|
| **The Medical Profession and a Ministry of Health** | 303 |
| **The Speaker and the Hospitals** | 304 |
| **Hospital and Institutional News** | 305 |
| A Native General Hospital—Reopening of the Science Museum—Some Important Bequests—Officers at Bootham Park—The Retention of Indispensable Men—Sand-bagged Hospital Statues—Should Hospital Buildings be made Bomb-proof?—Disappearing Diseases—T.N.T. Poisoning—Busy Dentists—A Growing Cottage Hospital—Women Tuberculosis Officers—Tehidy Sanatorium—"May I Brag?"—Help Needed at Air-raid Shelters—Wood Fires—Voluntary Agencies in Lambeth—Johannesburg Babies—The Third London *Gazette*. | |
| **The Practice of Percussion** | 309 |
| As Influenced by the Newer Methods. | |
| **The Order of the British Empire** | 311 |
| **Physical Culture in Education** | 312 |
| I. Greek, French, and German Systems. | |
| **How Not to Run a War Hospital** | 313 |
| Institutional Housekeeping Under the Poor-Law. | |
| **Income by Hundredweight** | 314 |
| The Organisation of Gifts in Kind. | |
| **The Editor's Letter-Box** | 315 |
| The Shortage of Doctors. | |
| **Hospital and Institutional Results** | 315 |
| **Nursing Affairs in Ireland** | 316 |
| What Every Trained Nurse Should Know. | |
| **The Matrons' and Sisters' Department** | 317 |
| Nursing Progress and its Developments: V. The Nurse-Pupil's First Stage. | |
| Round the Hospitals. | |
| Queen Alexandra Dame Grand Cross—Mr. J. G. Wainwright and His Nurses—The College Election—Mrs. May Webster a Dame Commander—Good Fortune for Trained Nurses—Press Unanimous for Nation's Gift. | |
| **Matrons' and Sisters' Appointments** | 320 |
| **The College of Nursing** | 321 |
| Hundreds of Nurses Join its Register. | |
| **Our Hospitals' Christmas, 1917** | 322 |
| Bolo Buried and Forgotten Everywhere. | |
| **The Institutional Worker** | (Suppl.) 1 |
| The Equipment of an Out-Patient Department: Answer to November Question. | |
| Enquiries and Answers. | |
| Employment and Training | |
| Miscellaneous. | |

